# Twenty-five years of observations of soil organic carbon in Swiss croplands showing stability overall but with some divergent trends

**DOI:** 10.1007/s10661-019-7435-y

**Published:** 2019-04-13

**Authors:** Andreas Gubler, Daniel Wächter, Peter Schwab, Michael Müller, Armin Keller

**Affiliations:** Agroscope, Swiss Soil Monitoring Network NABO, Reckenholzstr. 191, 8046 Zurich, Switzerland

**Keywords:** Soil organic carbon (SOC), Soil monitoring, Minimum detectable change (MDC), Agricultural management

## Abstract

The temporal evolution of soil organic carbon (SOC) is of major importance given its status as a key parameter in many soil functions. Furthermore, soils constitute an important reservoir of carbon in our environment. In light of climate change, consistent SOC data over extended periods in combination with information on agricultural management are much required, but still scarce. We report SOC changes in the topsoil (0–20 cm) of Swiss cropland measured at well-defined monitoring sites resampled every 5 years from 1990 to 2014 by the Swiss Soil Monitoring Network NABO using consistent sampling protocols and quality assurance. Data on agricultural management practices were retrieved from farmers. Overall, SOC remained stable for the ensemble of monitoring sites, although increasing and decreasing trends were observed for individual sites, ranging from − 11 to + 16% relative change per decade. Changes in the agricultural management of cropland triggered substantial changes in SOC contents for some sites. Moreover, sites with a low ratio of SOC/clay (< 0.1) generally showed more positive trends than sites with higher ratios. We presume that SOC was either at or near steady state, given the consistency of management practices over the last few decades. Finally, our study provides insights into the uncertainties related to (real-world) SOC monitoring and underlines the relevance of short-term SOC variations that could hamper the detection of long-term trends. The minimum detectable change (MDC) by the applied monitoring scheme is estimated at 0.35% per year, in relative terms.

**Electronic supplementary material:**

The online version of this article (10.1007/s10661-019-7435-y) contains supplementary material, which is available to authorized users.

## Introduction

Soil organic carbon (SOC) is a key property affecting the quality and many functions of soil, including the filtering of pollutants, the cycling and storage of nutrients and water and soil fertility (Bünemann et al. 2018). In addition, the interactions between climate change and terrestrial carbon pools have been widely discussed over the last two decades, particularly the role of soils as potential sinks or sources (Eglin et al. 2010; Mackey et al. 2013; Read et al. 2001). Globally, soils store roughly 1550 Gt of organic carbon and have the potential to sequester 0.4–1.2 Gt C year^−1^, with 0.4–0.8 Gt C year^−1^ related to cropland soils (Lal 2004a). Accordingly, the potential effects of agricultural practices on SOC are of major interest.

It is commonly recognised that agricultural management affects SOC. Important factors include (no-)tillage practices, mulching, nutrient management, crop rotation, inclusion of cover crops and erosion control (Lal 2004b). For example, application of straw or manure tends to increase SOC sequestration compared with practices that use only mineral fertilisers (Han et al. 2016). Equally, organically farmed soils have higher SOC concentrations and stocks, attributed to differences in external C inputs and crop rotations (Gattinger et al. 2012). Furthermore, the presence of permanent or temporary grasslands is generally associated with increased SOC (e.g. Guo and Gifford 2002), although these differences might, to some extent, arise from correlations with other factors such as altitude, climate, soil properties and inputs of manure and biomass. In addition, any differences might be limited to topsoils; SOC stocks of entire soils were similar, e.g. for cropland vs. grassland (Don et al. 2009) and tillage vs. no-till (Martínez et al. 2016), the systems only differing in their distributions over depth. However, consistent data on SOC over extended periods under real-world conditions, including related information on agricultural management practices, are still scarce.

Over the last few decades, various studies on the evolution of SOC have been undertaken at regional to national scales. In this context, one may distinguish two types of investigation: comparisons of paired observations from resampling of soils at the same sites at different times (e.g. Capriel 2013; Heikkinen et al. 2013; Taghizadeh-Toosi et al. 2014) and studies using soil data from sampling campaigns at different sites and times (unpaired, e.g. Lettens et al. 2005; Poeplau et al. 2015). In addition, many long-term field trials also assess SOC (for instance, see BonaRes-Data Centre 2017), but these usually reflect specific treatments regarding fertilisation and cropping systems, which do not necessarily represent common practice.

When assessing carbon stocks for whole regions, the unpaired sampling approach seems more straightforward given that soil sampling, sample preparation and analytical methods are comparable for different periods. Such approaches provide integration both over environmental processes (e.g. climate) and socio-economic drivers (e.g. shifts in land use). For instance, Poeplau et al. (2015) reported increasing SOC in Sweden due to increasing proportions of grassland, which was in turn triggered by the increased popularity of horses. In contrast, the resampling of a set of well-defined monitoring sites is the most efficient approach for assessing long-term effects of climate and agricultural management on SOC for a given land use (Lark 2009). Some studies based on resampling of the same sites showed slightly positive or negative trends overall (e.g. Steinmann et al. 2016; Heikkinen et al. 2013; Riley and Bakkegard 2006), while others found non-uniform evolutions with increasing, constant and decreasing SOC trends at individual sites (e.g. Hanegraaf et al. 2009; Taghizadeh-Toosi et al. 2014; see “Results and discussion” section).

For the present study, we assessed topsoil (0–20 cm) SOC of croplands using samples collected from 1990 to 2014 at five-yearly intervals at 30 long-term monitoring sites from the Swiss Soil Monitoring Network (NABO). The main objective was to assess the long-term evolution of SOC contents at cropland sites. More specifically, we addressed the following research hypotheses: (i) changes in the agricultural management of cropland trigger substantial changes in SOC and (ii) the temporal evolution of SOC is influenced by the presence of meadow (temporary grassland) within the crop rotation, with more positive trends for higher than for lower proportions of meadow.

## Materials and methods

### Long-term monitoring sites

**Fig. 1:**
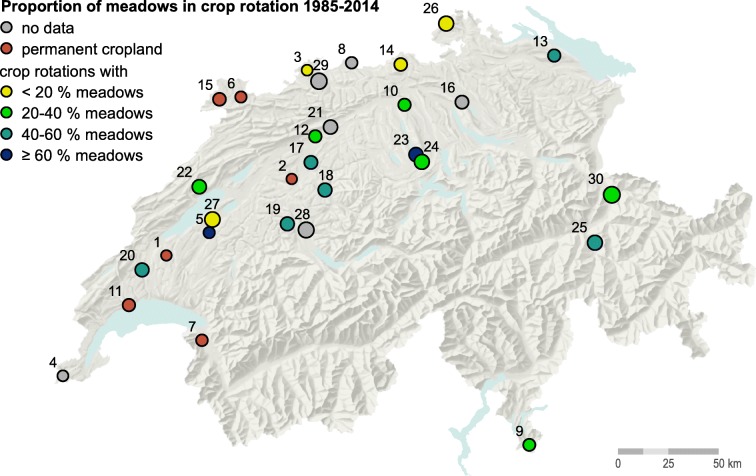
Selected cropland monitoring sites of the Swiss Soil Monitoring Network (NABO) and their proportion of meadows in crop rotation. Symbol sizes are proportional to the (log of the) mean organic carbon content of topsoil (0–20 cm). The labels indicate the site IDs

NABO operates about 100 long-term monitoring sites throughout Switzerland (Gubler et al. 2015), 30 of which are used as permanent cropland or cropland-meadow rotation and considered here (Fig. 1). Throughout this study, we use the term ‘meadow’ for temporary grassland. In the Swiss context, these are typically grass-clover leys grown for 1 to 5 years within a crop rotation.

Swiss croplands cover 4070 km^2^ (FSO 2017), implying roughly one monitoring site for every 140 km^2^. The soil conditions and agricultural management practices were representative of the variability of Swiss cropland. However, the sites cannot be considered representative with regard to agricultural practice, soil type and climatic conditions for Swiss agro-ecosystems in a statistical sense, given that they were selected non-randomly in the mid-1980s to monitor heavy metal pollution in soils. From 1990 to 2014, the topsoils (0–20 cm) were sampled five times at five-yearly intervals, usually in spring. For each sampling, four replicates (each being a bulked sample of 25 sub-samples) were collected from the same precisely located area of 10 × 10 m^2^ using a stratified random sampling design (see Supporting Information [SI] SI.1 for details). In principle, SOC contents were also available for samples collected during a former sampling campaign (1985–1989), but these samples were collected substantially later in the year. The effect of the time of sampling may be substantial due to seasonal SOC patterns (e.g. Leinweber et al. 1994); these samples were therefore omitted to prevent artefacts.

The studied sites contained mineral topsoils with SOC ranging roughly from 10 to 40 g kg^−1^, pH_CaCl2_ from 5.1 to 7.5 and the fractions of clay and silt from 6 to 59% and 8 to 69%, respectively (Table 1). For 24 sites, management data on the cultivated main crops (cover crops were not recorded) and the inputs of farmyard manure were available from 1985 to date, according to farmers’ declarations (Franzen et al. 2017; Keller et al. 2005). Crops were grouped into meadows (temporary grassland), cereals (wheat, barley, rye and similar), so-called hoe crops (maize, potatoes, beets, rape) and other (e.g. vegetables). The proportions of these groups varied considerably between sites (Table 1, Figs. S2–S3). Altitude was correlated positively with the proportion of meadows and negatively with cereals. Mean annual inputs per site ranged from 0 to 5700 kg dry matter (d.m.) ha^−1^ year^−1^ for solid manure and from 0 to 2130 kg d.m. ha^−1^ year^−1^ for liquid manure (slurry). Manure inputs correlated positively with the proportion of meadows and negatively with cereals and hoe crops. The proportions of crops and manure inputs were considered as proxy variables reflecting agricultural management.

**Table 1:** Site characteristics and selected proxy variables reflecting the agricultural management for the cropland monitoring sites (ordered according to mean organic carbon content). Soil properties are indicated for the top 20 cm of the soils; clay and silt contents of the fine earth (< 2 mm) were analysed in the first sampling campaign only, pH values represent mean values over all sampling campaigns, and apparent density (AD) represents mean values of sampling campaigns 5 and 6. Management data represent mean values for 1985–2014 with mean values for two subperiods (1985–1999/2000–2014) in brackets (n.a.: management data not available)

Site	Altitude	pH	Clay^a^	Silt^a^	AD^b^	SOC	stock^c^	Manure application (kg d.m. ha^−1^ year^−1^)		Cultivated crops (%)^d^		
	m.a.s.l.	(CaCl_2_)	%	%	g cm^−3^	g kg^−1^	t ha^−1^	liquid (slurry)^e^	Solid^e^	Meadows^e^	Cereals^e^	Hoe crops^e,f^
1	684	5.2	16	38	1.21	11.9	29	0 (0/0)	0 (0/0)	0 (0/0)	61 (67/56)	39 (33/44)
2	557	6.0	12	35	1.30	12.0	31	640 (790/500)	590 (360/810)	0 (0/0)	32 (33/31)	61 (67/56)
3	324	5.3	15	72	1.46	12.8	38	950 (530/1370)	470 (750/190)	9 (10/7)	21 (40/0)	12 (23/0)
4	428	5.3	17	25	0.83	13.2	22	n.a.	n.a.	n.a.	n.a.	n.a.
5	488	6.4	15	16	1.38	14.4	40	900 (1130/670)	250 (490/0)	60 (40/80)	20 (33/7)	20 (27/13)
6	482	5.7	16	67	1.24	13.7	34	220 (200/240)	1010 (1120/890)	0 (0/0)	71 (73/70)	29 (27/30)
7	379	7.2	6	60	1.14	14.8	34	0 (0/0)	90 (0/180)	0 (0/0)	41 (42/40)	59 (58/60)
8	343	5.8	15	46	1.33	15.2	40	n.a.	n.a.	n.a.	n.a.	n.a.
9	336	5.9	21	38	1.19	15.5	37	70 (0/140)	2550 (2280/2810)	22 (25/20)	26 (33/20)	52 (42/60)
10	417	5.3	14	33	1.04	16.5	34	1010 (2020/0)	550 (820/280)	22 (46/0)	15 (8/21)	63 (46/79)
11	435	6.1	19	45	1.27	17.5	45	0 (0/0)	520 (690/350)	0 (0/0)	55 (58/53)	45 (42/47)
12	455	5.3	17	23	1.07	17.2	37	1780 (0/3560)	800 (860/740)	37 (0/47)	37 (50/33)	26 (50/20)
13	559	6.2	24	44	1.21	17.8	43	990 (980/1000)	2990 (2460/3510)	41 (42/40)	39 (46/33)	20 (12/27)
14	465	5.2	14	34	1.09	19.1	42	330 (500/160)	1510 (1940/1090)	18 (42/0)	43 (25/56)	39 (33/44)
15	538	5.4	26	67	1.18	18.8	44	340 (100/570)	1170 (1560/780)	0 (0/0)	59 (67/53)	41 (33/47)
16	440	7.2	10	23	1.01	19.9	40	n.a.	n.a.	n.a.	n.a.	n.a.
17	478	5.9	28	23	1.26	20.7	52	1620 (1750/1490)	810 (830/790)	48 (36/60)	24 (29/20)	28 (36/20)
18	618	5.1	18	38	1.13	21.6	49	1740 (1410/2070)	0 (0/0)	52 (58/47)	30 (25/33)	19 (17/20)
19	945	5.1	14	20	1.02	21.5	44	2130 (1840/2430)	5690 (9480/1910)	57 (54/60)	24 (29/20)	19 (18/20)
20	515	6.5	24	24	1.07	21.7	46	760 (690/830)	590 (1180/0)	43 (21/62)	40 (43/38)	17 (36/0)
21	450	5.4	36	45	1.23	23.7	58	n.a.	n.a.	n.a.	n.a.	n.a.
22	770	5.9	21	18	1.08	23.1	50	720 (820/630)	970 (840/1110)	26 (27/25)	48 (47/50)	26 (27/25)
23	500	5.8	26	36	1.07	23.6	51	2030 (1330/2730)	1570 (1600/1550)	66 (36/93)	14 (29/0)	17 (29/7)
24	450	5.6	31	31	1.11	27.1	60	2000 (2620/1380)	100 (0/190)	39 (50/29)	25 (14/36)	36 (36/36)
25	830	6.8	18	51	1.13	28.3	64	660 (580/750)	3630 (2200/5060)	48 (42/53)	30 (33/27)	22 (25/20)
26	545	7.2	59	30	0.97	28.9	56	1510 (1730/1300)	1240 (1610/870)	13 (0/25)	30 (43/19)	47 (57/38)
27	439	7.5	43	46	1.06	29.7	63	390 (670/120)	1190 (380/1990)	7 (0/12)	36 (42/31)	57 (58/56)
28	534	7.3	22	38	0.95	33.7	64	n.a.	n.a.	n.a.	n.a.	n.a.
29	626	7.1	41	46	0.78	36.8	58	n.a.	n.a.	n.a.	n.a.	n.a.
30	532	7.2	37	47	1.05	38.2	80	910 (640/1170)	1520 (1780/1270)	25 (25/25)	32 (33/31)	43 (42/44)

^a^Clay: particles < 2 μm; silt: 2–50 μm; sand + silt + clay = 100%

^b^Apparent density, defined as mass of fine earth (< 2 mm) per total soil volume (including stones, pores, etc.)

^c^Organic carbon stock for 0–20 cm soil depth; stock = SOC · AD · soil depth

^d^Percentage of each category relative to the total of years

^e^Mean values 1985–2014 with mean values for sub-periods (1985–1999/2000–2014) in brackets

^f^Hoe crops include maize, potatoes, beets, rape

### SOC analyses

SOC analyses were conducted using archived samples originally crushed, sieved and oven-dried at 40 °C. The archived samples were mixed well using a Turbula shaker prior to taking subsamples for SOC analysis. A further subsample was dried at 105 °C to correct measured SOC contents for remaining water in order to obtain SOC contents relative to dry matter of soil. Apart from standard operation protocols, further measures ensured the quality of the chemical analyses. Various reference soils with known SOC contents (covering the expected range) were measured within every batch to ensure the stability of the analytical system.

SOC content was determined either by wet oxidation (WO) and retitration of potassium dichromate (Swiss Standard Method; FAL 1996) or dry combustion (DC) with a CN-analyser (LECO TrueSpec CN) and subtraction of inorganic carbon where appropriate. Inorganic carbon was determined by digestion with hydrochloric acid and volumetric metering of the CO_2_ produced (FAL 1996). The WO method yielded lower SOC values than DC (Gubler et al. 2018). Relative recoveries of WO/DC ranged from 77 to 90% with median: 85%. Therefore, results of the WO method were recalculated to the level of DC using site-specific conversion factors (for details of the data harmonisation, see Gubler et al. 2018).

### Statistical analyses

All statistical analyses were based on log-transformed SOC contents to achieve constant variance over the whole range. If not stated otherwise, the mean of the four replicates per site and sampling was used. Two sites (5, 23) were converted into permanent grassland in around 2005 and were omitted when assessing temporal trends.

We assessed the site-specific SOC and the impact of site characteristics and management on this by deriving Spearman’s rank correlation coefficients from the mean values over all sampling campaigns. Additionally, we conducted a robust principal component analysis (ROBPCA implemented in R package rrcov; Hubert et al. 2005; Todorov and Filzmoser 2009) to assess the relationship between individual variables. For the ROBPCA, the original data were transformed as follows: log transformation for SOC contents and altitude, log transformation with an offset for inputs of farmyard manure (*x*^′^ = log(*x* + *c*) where *c* = *median*(*x*)/(*median*(*x*)/*q*_0.25_(*x*))^2.9^, an estimate for the 0.025 quantile), and arcsine square root transformation for the contents of clay and silt and the proportion of meadows, cereals and hoe crops. Soil pH and the ratio of SOC/clay were not transformed.

We used linear-mixed models (function lme of package nlme; Pinheiro et al. 2016) to assess the global evolution of SOC over time. Residual analyses according to Gubler (2017) revealed that the errors between individual samplings were independent. First, a model according to Eq. 1 was adapted to test for differences between individual sampling campaigns *t*.

1 $$ \log \left({\mathrm{SOC}}_{i,t}\right)={y}_{i,t}=\mu +{\alpha}_i+{\beta}_t+{\varepsilon}_{i,t} $$

Hence, *y*_*i,t*_, the (natural) logarithm of the SOC content of site *i* for sampling campaign *t*, was estimated by the overall mean *μ*, with a random intercept term *α*_*i*_ allowing for an individual SOC level per site, and a factorial variable *β*_*t*_ representing the levels of the individual sampling campaigns. The residuals are denoted by *ε*_*i,t*_. Second, we tested for a linear trend by adapting a model according to Eq. 2.

2 $$ \log \left({\mathrm{SOC}}_{i,t}\right)={y}_{i,t}=\mu +{\alpha}_i+\left(\beta +{\beta}_i\right)\cdotp t+{\varepsilon}_{i,t} $$

In this case, the temporal evolution was modelled using *(β + β*_*i*_*)·t*, where *β* represents the global linear trend and the random slope term *β*_*i*_ allows for a site-specific slope. In addition, we assessed the minimum detectable change (MDC) of the NABO monitoring scheme by conducting power analyses for simulated datasets, see SI.3 for details.

Finally, we estimated the uncertainty of the linear trends for each site using a hierarchical bootstrapping approach. For every site and sampling campaign, a bootstrap sample of size four was drawn from the (usually four) replicate soil samples available for the respective site and campaign. Based on the selected replicates, the mean of log(SOC) per site and sampling was calculated, and for every site, the slope of the linear regression was derived. In addition, the slopes were recalculated for the same data by omitting repeatedly the data from individual sampling campaigns to account for the effect of the single time points. The whole procedure was repeated in 500 iterations. Finally, for each site, the mean, median, as well as the 0.025 and 0.975 quantiles were derived from the ensemble of slopes and considered to be the probable range of the real trend. We are aware that, with four replicates per site and sampling, the number of bootstrap samples is limited. However, by combining the bootstrapping with repeated deletion of individual sampling campaigns, the variability of both levels (within and between samplings) can be captured. We compared the slopes of the individual sites with site characteristics and management data. For the latter, we considered the mean values from 1985 to 2014 as well as the differences for 1985–1999 vs. 2001–2014.

## Results and discussion

### Levels of SOC in Swiss cropland

**Fig. 2:**
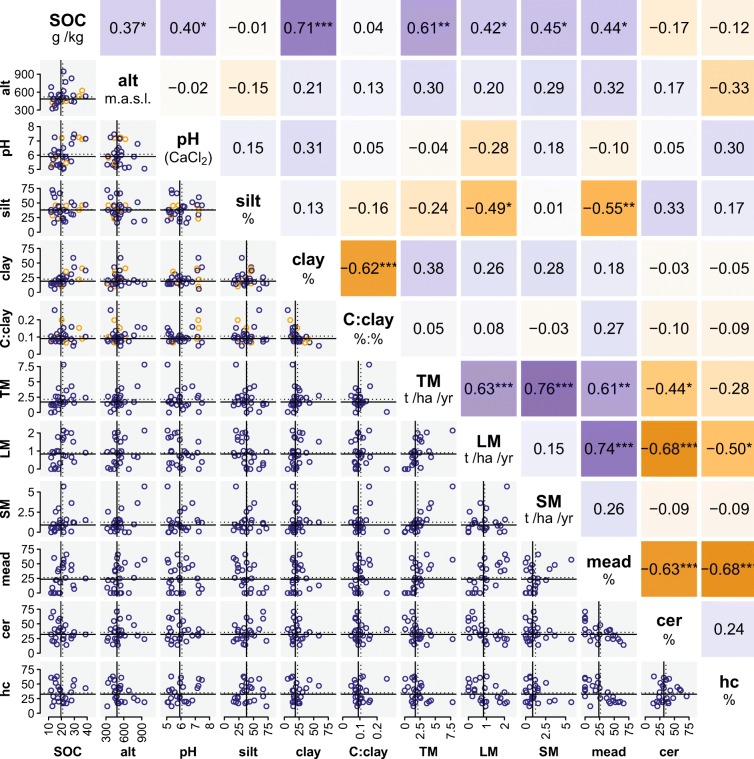
Site characteristics and proxies for agricultural management of the investigated sites (mean values of all sampling campaigns): soil organic carbon content (SOC, g kg^−1^), altitude (alt, metres above sea level), pH (CaCl_2_), contents of clay and silt (% of fine earth), ratio of SOC/clay (C/clay; % %^−1^), mean annual inputs of farmyard manure in total (TM; t dry matter ha^−1^ year^−1^) and for solid (SM) and liquid (LM) manure separately, and percentages of years (1985–2014) featuring meadows (mead), cereals (cer) and so-called hoe crops (hc; includes maize, rape, beets and potatoes) as main crop, lower panel: scatter plots with blue symbols representing sites with information on agricultural management (*N* = 24) and orange symbols representing sites without management data (*N* = 6). The broken lines indicate the means, and the solid lines indicate the median of all sites. Upper panel: Spearman’s rank correlation coefficients for pairwise complete observations. The stars indicate significant correlations (*p < 0.05; **p < 0.01; ***p < 0.001). Background colours indicate the degree of correlation

SOC levels largely seem to be governed both by soil properties, namely clay content, and agricultural management, namely farmyard manure input and crop rotation. Elevated clay contents, higher input of farmyard manure and higher proportions of meadows in the crop rotation coincided with higher contents of SOC (Fig. 2). However, most explanatory variables were strongly interrelated. For instance, altitude was correlated positively with clay content and the proportion of meadows, the latter strongly influencing the input of farmyard manure. Due to the very strong collinearities between the mentioned auxiliary parameters, we cannot properly disentangle the impact of site characteristics and agricultural management on SOC contents. Principal component analysis revealed very close relationships between SOC, total manure input and clay content, whereas altitude and the proportion of meadows showed slightly weaker correlations with SOC content (c.f. biplots in Fig. S4). Therefore, we concluded that the amount of farmyard manure was the key management factor for SOC levels. Furthermore, we suggest that the correlation between the proportion of meadows and SOC observed in this study and previous works (e.g. Guo and Gifford 2002) mainly arises from the associated changes in farmyard manure input, and only to a minor extent from the proportion of meadows. As stated above, we cannot clearly distinguish the effects of these two factors. However, our conclusion is supported by experimental long-term trials (e.g. De Bruijn et al. 2012). This hypothesis will be further discussed in the next section.

Clay content represented the main soil parameter governing SOC, while silt content showed no significant correlation with it. It is commonly recognised that clayey soils tend to store more SOC than sandy soils (Dexter et al. 2008), and indicators such as the ratio of SOC/clay have been suggested to evaluate the structural quality of soils (Johannes et al. 2017). Following the recommendations by Johannes et al. (2017), we applied a SOC/clay ratio of 0.12 as threshold between good and medium structural quality of soils (the slightly different threshold compared with the original publication arises from the higher SOC recovery of the method used here^1^). Most of the cropland monitoring sites of our study showed SOC/clay ratios approaching 0.12, except for soils low in clay showing ratios above 0.2 and very clayey soils showing ratios substantially lower. The ratio of SOC/clay seemed mostly independent of the other soil properties and agricultural management. Principal component analysis revealed only a weak link with altitude.

### Evolution of SOC over 25 years

**Fig. 3:**
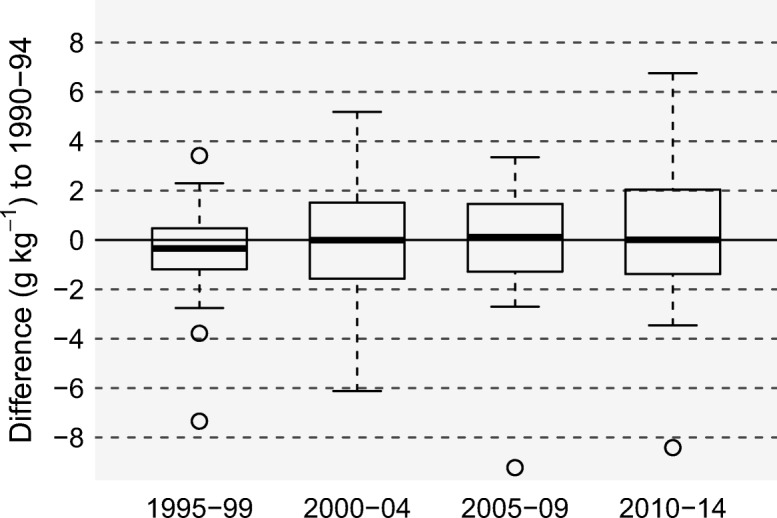
Soil organic carbon (SOC) in the top 20 cm of cropland sites (*N* = 28) across Switzerland: boxplot of differences (per site) in SOC between the individual sampling campaigns and the baseline 1990–1994

As far as the general evolution of SOC for all monitoring sites is concerned, only slight differences were observed between individual sampling campaigns (Fig. 3). On average, SOC contents were lowest in 1995–1999 and highest in the most recent campaign of 2010–2014. The estimates and 95% confidence intervals (CI; in brackets) from the linear-mixed model (Eq. 1) were 19.6 (16.7–23.1) g kg^−1^ for the 1995–1999 campaign and 20.1 (17.1–23.7) g kg^−1^ for the last campaign. The differences between sampling campaigns were statistically non-significant for all combinations. In addition, there was no linear trend either over the period as a whole or for the shorter period 1995–2014; the respective linear-mixed model (Eq. 2) did not indicate any significant trend (*p* » 0.05).

**Fig. 4:**
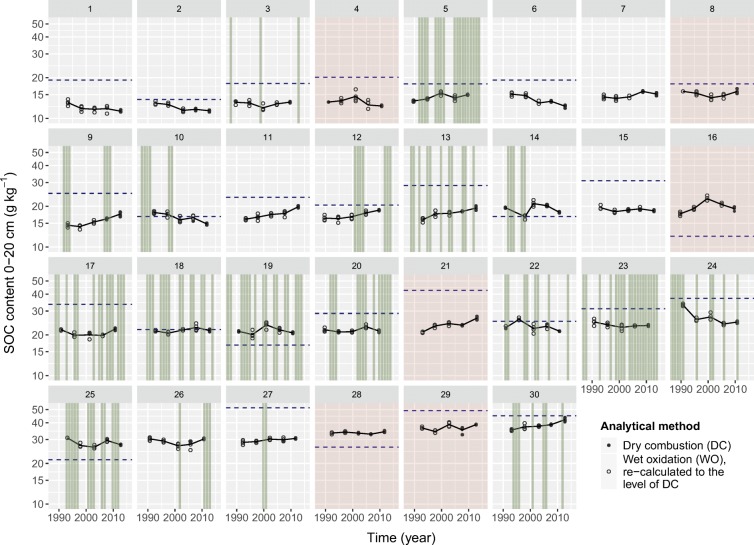
Evolution of soil organic carbon (SOC) contents in topsoils (0–20 cm) for 30 cropland sites. Broken lines indicate the SOC content where the ratio of SOC/clay equals 0.12 (corresponding SOC/clay ratios at site 7 and 26 are outside the plotted range and were 7 g kg^−1^ and 71 g kg^−1^, respectively). Circles indicate SOC contents of individual replicates; solid lines indicate the means of all replicates per sampling. Green vertical lines indicate years with temporary grassland (meadows), reddish panels indicate sites without information on cultivated crops

**Fig. 5:**
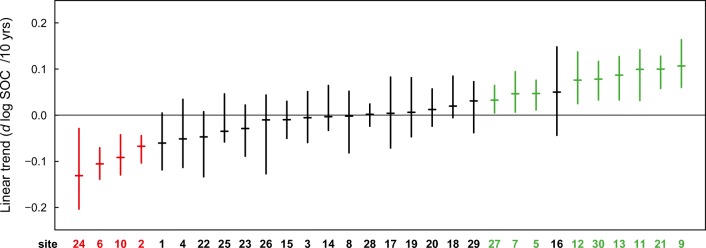
Slopes of linear trends of soil organic carbon (SOC) contents for individual sites: horizontal lines indicate the median and vertical lines the 0.025–0.975 percentile range of the bootstrap population. Trends are indicated as change per 10 years of log-transformed SOC contents

Despite these findings, we nevertheless observed significant linear trends at site level (Fig. 4). There were 4 sites with declining trends, 9 sites with increasing trends, and 17 sites with stable or indistinct trends from 1990–1994 to the 2010–2014 sampling campaign (Fig. 5). The linear trends ranged from − 0.13 to + 0.11 log(SOC) per decade corresponding to relative SOC changes ranging from − 12 to + 11% over 10 years. We found no relationship between the magnitude of the trends and most site characteristics (Fig. S5) except for the initial ratio of SOC/clay. There was a tendency for sites with a low SOC/clay ratio at the beginning of the time series (roughly below 0.1) to increase in SOC over subsequent decades, while for sites with higher initial ratios, reductions in SOC were observed more frequently.

Various authors have been successful in linking soil properties with temporal changes in SOC. For England and Wales, Bellamy et al. (2005) found that initial SOC contents governed the direction of the trends. They observed that very low initial contents coincided with increasing trends of SOC, whereas high initial contents led to decreases. Similar results were reported by Hanegraaf et al. (2009) for Dutch soils, who nonetheless concluded that initial SOC content “need not be the driver per se, and that other factors such as land use and soil management may be of influence as well”. Our study suggests that the initial ratio of SOC/clay, rather than the initial SOC content, determined the SOC trends at the investigated sites. We assume that there is a specific optimum ratio of SOC/clay for any soil. Thus, soils with low ratios (presumably indicating a deteriorated soil quality) have a potential to increase SOC substantially by adjusting the agricultural management.

Our findings for 30 cropland monitoring sites in Switzerland are in general agreement with published data. Table 2 summarises European studies based on field data, all of which (i) covered a period starting around 1980 or later, (ii) included at least two time points and (iii) were at regional scale or larger (field trials were not included). Of studies based on resampled sites, four reported generally declining SOC in cropland, namely Finland (Heikkinen et al. 2013), Scotland (Chapman et al. 2013), southeast Norway (Riley and Bakkegard 2006) and West Flanders (Belgium; Sleutel et al. 2006). However, for Scottish soils, declining SOC concentrations coincided with increasing depths of plough layers; hence, the decreases resulted from dilution of topsoil with subsoil, whereas SOC stocks remained stable. For the Cologne-Bonn region (Germany), increasing topsoil contents coincided with decreasing subsoil contents and decreasing bulk densities (Steinmann et al. 2016). Finally, four reports showed sites with increasing, constant and decreasing contents, namely Bavaria (Germany; Capriel 2013), Denmark (Taghizadeh-Toosi et al. 2014), England and Wales (Bellamy et al. 2005) and the Netherlands (Hanegraaf et al. 2009).

**Table 2:** Compilation of studies assessing the temporal evolution of soil organic carbon (SOC) concentrations and/or stocks of European croplands at regional or larger scale covering the period from around 1980 to present. Only studies including at least two time points are listed. Where possible, results for cropland topsoils were extracted and recalculated to give mean annual changes. In the case of multiple studies for identical areas, we considered the most recent

Location	Purpose^q^	Land use^r^	Period	Time points	Design	Relocation accuracy	Sampled depth	Sampled area	# of sub-samples	Analytical method^v^	QA of lab^z^	Main findings for cropland^aa^		
						(m)	(cm)	(m × m)				conc	stocks	Summary
Switzerland^a^	M	C	1985 to 2014	6	paired	< 0.2	0–20	10 × 10	4 × 25	WO, DC^w^	C & R	**≈**		no uniform trends, sites with increasing, stable, and decreasing contents
Bavaria (Germany)^b^	M	C, G	1986 to 2007	4	paired	high	0–15	1000	4 × 25	DC	n.s.	**≈**		no uniform trends, sites with increasing (19 sites), stable (50), and decreasing contents (23)
Cologne-Bonn region (Germany)^c^	R	C	2005 vs. 2013	2	paired	< 2–3	0–60 (0–37, 37–60)	(50 m transect)	10	DC	C & R	**+**	**–**	contents increasing in topsoils (+ 0.05 g kg^−1^ year^−1^) and decreasing in subsoils (− 0.08 g kg^−1^ year ^−1^), but generally decreasing stocks due to lower bulk densities in second sampling (− 0.56 t ha^−1^ year^−1^ for 0–60 cm)
Denmark^d^	M	C	1986 to 2009	3	paired	< 40	0–25, 25–50, 50–100	50 × 50	16	DC	C		**≈**	no uniform trends, sites with increasing, stable, and decreasing contents
England & Wales^e^	M	all	1978–83 vs. 1994–96	2	paired	20–50	0–15	20 × 20	25	WO, LOI^x^	R	**≈**		increases for initial SOC < 20 g kg^−1^ (+ 0.3 g kg^−1^ year^−1^), no trend for 20–30 g kg^−1^, decreases for 30–50 g kg^−1^ (− 0.4 g kg^−1^ year^−1^), bigger losses for soils > 50 g kg^−1^
Finland^f^	M	C, G	1987 to 2009^r^	3^s^	paired	n.s.	0–15	10 × 10	4/10	DC	n.s.	**–**	**–**	overall decreasing trends for contents (− 4 g kg^−1^ year^−1^) and stocks (− 0.2 t ha^−1^ year^−1^)
Netherlands^g^	A	C, G	1984 to 2004	4–5	paired	n.s.	0–25	field (~ 20 ha)	40	WO, DC, LOI^x,y^	C	**≈**		no uniform trends, sites with increasing, stable, and decreasing contents
Scotland^h^	M	all	1978–88 vs. 2007–09	2	paired	n.s.	0–75 to 0–100	(from soil profile)	–	DC	S	**–**	**0**	concentration decrease from 40.3 to 37.0 g kg^−1^ for plough layer coinciding with plough layers gaining thickness (dilution effect), no significant change for stocks 0–15 and 0–100 cm
Southeast Norway^i^	M	C	1990–93 vs. 2001	2	paired	n.s.	0–25	(100 m transect)	15	LOI (SOM)	n.s.	**–**		overall decrease by − 0.04% SOM year^−1^ (range for different regions: 0 to − 0.09% SOM year^−1^)
West Flanders (Belgium)^j^	M	C	1989–94 vs. 2003–04	2	paired	10	plough layer (≈ 0–32)	≈ 50 (4 m radius)	6	DC	n.s.	**–**	**–**	decreasing trends for contents (− 0.32 g^−1^ year^−1^) and stocks (− 0.19 t ha^−1^ year^−1^) correlating with decreasing OC inputs through manure and straw
Belgium^k^	A, R	C	1990 to 2000	2	un-paired		0–15 or 0–23^u^	n.s.	n.s.	WO, LOI^x^	n.s.		**–**	overall decrease by − 0.2 t ha^−1^ year^−1^ for 0–20 and 0–30 cm, − 0.4 t ha^−1^ year^−1^ for 0–100 cm
Flanders (Belgium)^l^	A	C	1989 to 2000	4	un-paired		0–24	n.s.	n.s.	n.s.	n.s.		**–**	overall decrease by − 0.9 t ha^−1^ year^−1^ for 0–100 cm (extrapolated)
France^m^	A	C	1990–97 vs. 1998–2005	2	un-paired		plough layer	field	10–15	WO	n.s.	**≈**		no uniform trend, majority of regions (‘cantons’) showed no significant change, each 10–15% of the regions with increasing and decreasing contents, respectively
Franche-Comté (France)^n^	A	C, G	1990 to 2004	3	un-paired		plough layer	n.s.	10–15	WO	n.s.	**–**		overall decrease by − 0.26 g kg^−1^ year^−1^
Netherlands^o^	A	C	1984 to 2004	21^t^	un-paired		0–25	field (≈ 20 ha)	40	WO, DC, LOI^x,y^	C	**+**		slight increases over whole period for cropland (+ 0.08 g kg^−1^ year^−1^) and fields under maize (+ 0.23 g kg^−1^ year^−1^)
Sweden^p^	M	C, G	1988 to 2014	3	un-paired^u^		0–20	**≈** 80 (5 m radius)	9	DC	R, L	**+**		overall increase from 24.8 to 26.7 g kg^−1^ (≈+ 0.09 g kg^−1^ year^−1^) attributed to increasing proportions of grassland

^a^This study

^b^Capriel (2013)

^c^Steinmann et al. (2016)

^d^Taghizadeh-Toosi et al. (2014)

^e^Bellamy et al. (2005)

^f^Heikkinen et al. (2013)

^g^Hanegraaf et al. (2009)

^h^Chapman et al. (2013)

^i^Riley and Bakkegard (2006)

^j^Sleutel et al. (2006)

^k^Lettens et al. (2005)

^l^Sleutel et al. (2003)

^m^Chauveau et al. (2014)

^n^Saby et al. (2008a)

^o^Reijneveld et al. (2009)

^p^Poeplau et al. (2015)

^q^Purpose of sampling: M: monitoring and inventories, R: research; A: routine analyses for agriculture

^r^Assessed land uses: C: cropland, G: grassland (only results for cropland considered for this compilation)

^s^First sampling in 1974 not considered for this compilation

^t^Yearly

^u^Recalculated to 0–20, 0–30 and 0–100 cm

^v^WO: wet oxidation method, e.g. Walkley-Black, DC: dry combustion (CN analyser), LOI: loss on ignition

^w^WO results recalculated to level of DC

^x^LOI for carbon-rich soils

^y^WO until 1994, DC thereafter

^z^Quality assurance: measures to ensure validity and stability of OC analyses as reported in the respective publication, C: control samples/standards, R: reanalyses of samples, S: simultaneous analyses of samples, L: analyses by a single laboratory

^aa^Trend reported for cropland topsoils, + increase, 0 stable, − decrease, ≈ no uniform trend

*SOC* soil organic carbon, *SOM* soil organic matter

In general, reliable detection of long-term trends of SOC relies on consistent data from an adequate number of time points. When using data from two or three time points only (as for the majority of studies listed in Table 2), short-term variability can hardly be separated from long-term trends; this also holds for our data (Fig. 4) despite strict sampling protocols and quality assurance over the whole process chain. Over the short term, SOC fluctuates due to seasonal patterns and/or random processes (e.g. rainfall) regardless of the long-term evolution. Short-term variability of SOC has only been recognised by a few researchers: for instance, Leinweber et al. (1994) reported variations of up to 40 g SOC kg^−1^ (representing 15% relative change) within 1 year for a single field, and Wuest (2014) reported relative variations of 14–16% within 39 months. From a long-term perspective, short-term variability represents noise and hampers the detection of long-term trends. After five sampling campaigns (time points), a monitoring scheme according to NABO is able to detect a relative global change in SOC provided that it is at least 0.35% per year (c.f. SI3: Table S1, Fig. S8, Fig. S9). This roughly corresponds to a 7% relative change after 20 years (corresponding, e.g. to a mean increase from 20 to 21.4 g kg^−1^ SOC over two decades). However, to our best knowledge, only one previous study reported MDCs for monitoring schemes using SOC data from resampled sites: for England and Wales, the MDC for cropland was estimated at 2 g kg^−1^ using two time points separated by 12 years (Saby et al. 2008b). This corresponded roughly to a 7% relative change in total or 0.6% per year, in line with the MDCs reported in Table S1 (England and Wales: one site per 73 km^2^).

### Impact of agricultural management

The magnitude of SOC trends was correlated neither with the proportions of crop types in the crop rotation nor with the average inputs of animal manure over the whole period 1985–2014 (Fig. S6). Indeed, two sites (1 and 6) characterised by high proportions of cereals (> 50%), the absence of meadows and below-average inputs of farmyard manure showed decreasing SOC trends. However, other sites with comparable proportions of cereals and manure inputs showed trends that were stable (site 15) or even increasing (site 11). The positive trend for site 11 may be attributed to the no-till system applied. The management data for the remaining sites indicate that all of these were under tillage systems.

Shifts in agricultural management between the first (1985–1999) and second period (2000–2014) explain the observed trends to some extent (Fig. S7). For most of the investigated sites, increasing proportions of meadows correlated with increasing SOC contents. In addition, reducing the proportions of cereals and/or hoe crops was accompanied by increasing SOC. Furthermore, reduced inputs of liquid manure correlated with declining SOC. These observations were closely related in most cases because the changing proportions of meadows were associated with a change in manure inputs (c.f. sites 10, 12, 24 and 25). Increasing the proportion of meadow was generally compensated by decreasing the proportion of hoe crops (for sites 12, 17, 20, 26) or vice versa (site 10). Two sites were converted from cropland-meadow rotation to permanent grassland around 2005. While SOC seemed to increase slightly after the conversion at site 5, site 23 showed declining SOC prior to conversion, after which it stabilised (Fig. 4). Overall, our analysis of the changes in agricultural management confirmed our hypothesis that these trigger substantial changes in SOC.

While various authors have confirmed the major impact of agricultural management on SOC evolution (c.f. Table 2), specific management options may have different effects by region and inherent boundary conditions. For the Netherlands, there were more negative trends for permanent maize than for maize-grassland rotations (Hanegraaf et al. 2009). For Denmark, topsoil SOC was found to be positively correlated with the proportion of temporary grasslands in crop rotation, straw incorporation for autumn-sown crops and the application of cattle manure (Taghizadeh-Toosi et al. 2014). In contrast, pig manure, other organic manures and ploughing had no significant effects. Furthermore, as mentioned above, the presence of meadows within the crop rotation is generally associated with increased SOC (e.g. Guo and Gifford 2002), although Persson et al. (2008) showed that the inclusion of meadows in crop rotations positively influences SOC in some cases, but has no impact in others.

In light of the results from these studies, the lack of correlation between SOC trends and average agricultural management found in our own study needs further consideration. As reported by others, we expected more positive trends for higher than lower proportions of meadow or permanent cropland (our research hypothesis). However, for Swiss agro-ecosystems, crop rotations including various crop types (and typically also meadows) are well established and reflect common agricultural practice. Crop rotations are an important pillar of integrated production and organic farming to improve soil fertility and nutrient cycling and to reduce diseases and pests (Agroscope 2017; Charles et al. 2011). Therefore, most sites may have approached steady state, reflecting the agricultural management and crop rotation of past decades. This is supported by the observation that SOC changed at those sites where the management shifted substantially, i.e. where boundary conditions such as the farming system and fertilisation changed. Based on observations subsequent to changes in agricultural management, and the impact of management on SOC levels in general (c.f. first paragraph of ‘Results and discussion’ section), we conclude that the input of manure was the main driver for SOC changes in conjunction with the initial SOC/clay ratio. Thus, our findings confound to some extent the hypothesis that the presence of meadows within a crop rotation per se increases SOC. This idea is supported by De Bruijn et al. (2012) who showed that SOC evolution in grassland strongly depends on fertilisation; indeed, fertilisation regimes might explain the differing impact of meadows in crop rotations on SOC contents observed by Persson et al. (2008).

While shifts in management to some extent explain varying trends between sites, a substantial part of the variance remains unexplained. On the one hand, the number of sites is small considering the numerous variables, most of them inter-related. On the other hand, some important data, namely on cover crops and the intensity of soil working, were unavailable, not being recorded by farmers. Besides improving soil quality in general, cover crops are known to increase SOC stocks by 0.32 ± 0.08 Mg ha^−1^ year^−1^ for the top 22 cm of soil (Poeplau and Don 2015). The impact of soil working on SOC, particularly the benefit of no-tillage systems, is still unresolved. Generally, no-tillage increases topsoil SOC, although the SOC stock over the whole soil profile remains stable (Martínez et al. 2016). Hence, we assume that knowledge on cover crops and soil working might explain a substantial proportion of the remaining variance.

## Conclusions and perspectives

Using a paired soil-monitoring design with strict quality assurance, we generally found stable SOC contents over the last 25 years for monitoring sites in Swiss cropland. Due to the great variety of agricultural practices and soil properties, we observe increasing, stable and decreasing SOC trends at individual sites. These findings accord with comparable studies across Europe, but contradict some reports of a general SOC decline for cropland (c.f. Table 2 and discussion). Our long-term study clearly indicates that SOC contents in cropland have approached a steady-state at sites where agricultural management and crop rotation have remained relatively constant. Substantial changes in SOC were mostly observed to follow changes in agricultural management and crop rotation. We suggest that changes in the input of farmyard manure during the period of interest mainly induced these changes in SOC.

Furthermore, our data confirm the relevance of short-term variability of SOC (c.f. SI3), the magnitude of which relative to long-term trends presents a serious challenge for soil monitoring. Reliability and MDC may be substantially improved by increasing the number of resamplings per site. Furthermore, sound knowledge about the variability of soil bulk density and its impact on the effective sampling depth might further improve the detection of trends. Whenever possible, an approach using equivalent soil mass should be considered (e.g. as proposed by Wuest 2014). In contrast, long-term SOC trends are barely detectable when using as few as two or three sampling campaigns.

SOC is a key parameter in many soil functions. Future SOC evolution must therefore be systematically monitored, particularly because the climate is expected to become continuously warmer over the next few decades, with above-average increases for Switzerland (CH2011 2011), and because indications suggest that SOC sequestration will decrease markedly during exceptionally hot and dry European summers (Ciais et al. 2005). Investigations could also assess more systematically the impact of agricultural management options under real-world conditions. In addition, carbon stocks for entire soils should be addressed to distinguish changes in SOC distributions over depth from increases in total SOC stocks. Furthermore, SOC short-term variability and its consequences for soil monitoring should be considered more thoroughly. Finally, further reliable data are required to show SOC evolution for other regions and land uses.

## Electronic supplementary material

ESM 1 (PDF 2101 kb)

## Footnotes

1 Johannes et al. (2017) proposed a SOC:clay ratio of 1:10 = 0.1 as threshold between good and medium structural quality of soils. For SOC analyses, they used wet oxidation, whereas for our study we reported results of dry combustion. Since the first method systematically yields lower SOC contents compared with the latter, we adapted the SOC:clay ratio by accounting for the different SOC recovery. Median recovery of wet oxidation: 85% (c.f. ‘SOC analyses’). Hence, adapted ratio = 0.1: 0.85 ≈ 0.12.
