# Supplementary material for: Twenty-five years of observations of soil organic carbon in Swiss croplands showing stability overall but with some divergent trends
Source: Environ Monit Assess. 2019 Apr 13;191(5):277. doi: 10.1007/s10661-019-7435-y (PMC6469600; doi:10.1007/s10661-019-7435-y)
Supplement: Supplementary file 1 — (PDF 2101 kb) [file 10661_2019_7435_MOESM1_ESM.pdf]

Supplementary Information (SI) to

Twenty-five years of observations of soil organic carbon in Swiss croplands showing stability overall but with some divergent trends

Authors:

Andreas Gubler (1)

Daniel Wächter (1)

Peter Schwab (1)

Michael Müller (1)

Armin Keller (1)

(1) Agroscope, Swiss Soil Monitoring Network NABO, Reckenholzstr. 191, 8046 Zurich, Switzerland;

Corresponding author:

andreas.gubler@agroscope.admin.ch, +41 44 377 76 66

Contents

SI1: Monitoring sites and soil sampling 2

SI2: Additional Figures for Results & Discussion... 4

SI3: Minimum detectable change (MDC) and variability... 6

References 11

SI1: Monitoring sites and soil sampling

For each (re)sampling, four replicate samples were collected from the top 20 cm (fixed sampling depth measured from the soil surface). The replicates per time point, as well as all repeated samplings, represent the same area of 10 m x 10 m, accurately relocated using well documented reference points and buried magnets. The relocation accuracy was estimated at < 0.2 m. Each replicate consisted of 25 subsamples obtained using a gouge auger 2.5 cm in diameter according to a stratified random scheme: the sampled area was divided into 100 subplots of 1 m^2 with one subsample taken randomly within each subplot. Subsamples were bulked accordingly (Fig. S1; four replicates have an effective sampling support of 9 m x 9 m shifted by roughly 1 m for individual replicates). The soil samples were oven-dried at 40 °C and subsequently crushed and sieved to remove coarse soil components (> 2 mm diameter), before being archived in plastic containers. The whole process from sampling to lab analysis was standardised using standard operation protocols.

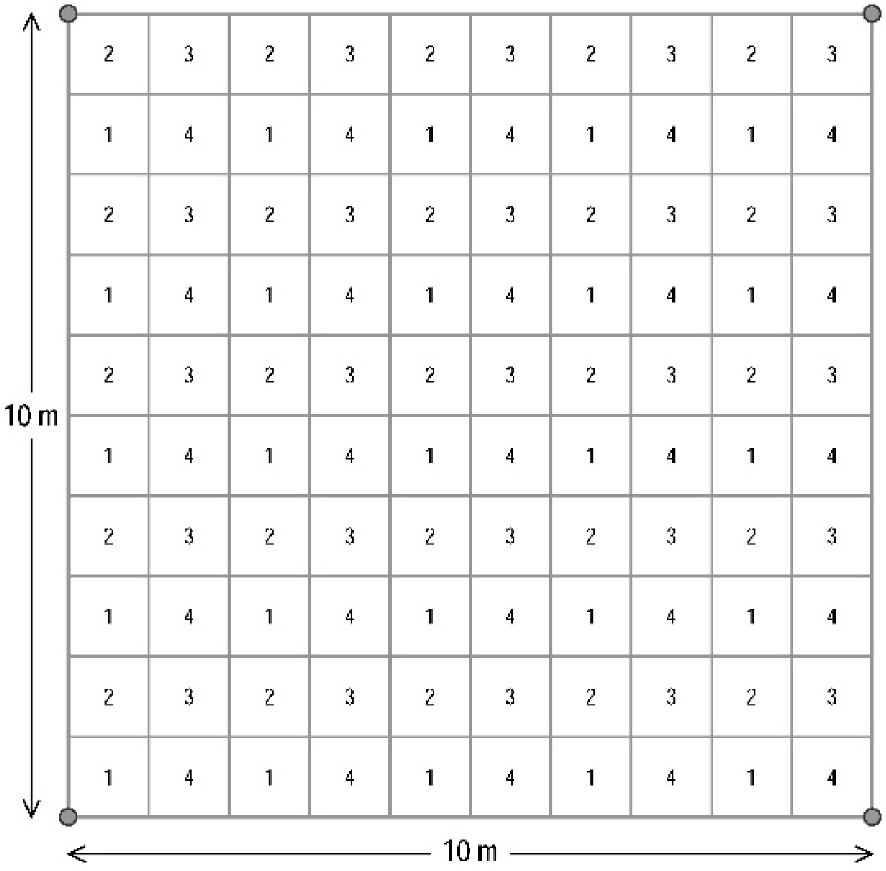

Fig. S1. Sampling protocol of Swiss Soil Monitoring Network NABO to collect composite samples 0-20 cm.

Site characteristics and available management data are summarised in Table 2 and Fig. S2. Differences in management between two periods (1985-1999 vs. 2000-2014) are illustrated in Fig. S3 and Fig. S4. For details of the presented data please consult the main text.

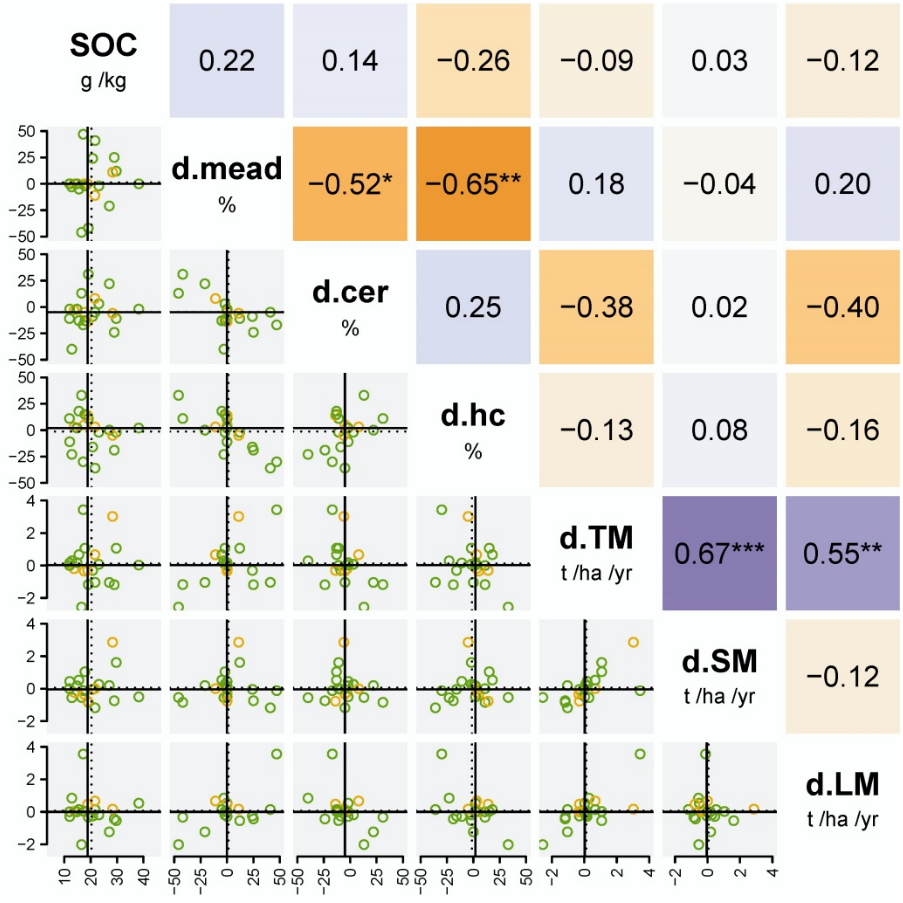

Fig. S2. Comparison of management data for the two periods 1985-99 (P1) vs. 2000-2014 (P2): soil organic carbon content (SOC, g kg^{-1}, mean of all sampling campaigns), differences P2-P1 in percentages of years featuring meadows (d.mead), cereals (d.cer), and so-called hoe crops (d.hc; includes maize, rape, beets, and potatoes) as main crop, and the differences P2-P1 in mean annual inputs of farmyard manure in total (TM; t dry matter ha^{-1} yr^{-1}) and for solid (d.SM) and liquid (d.LM) manure separately. Lower panel: scatter plots with orange symbols representing permanent cropland sites (N=6) and green symbols representing sites with cropland-meadow rotations (N=15). Site 17 was omitted from this plot due to its extreme changes in manure inputs (c.f. Table 2 in the main text). The broken lines indicate the means, and the solid lines indicate the median of all sites. Upper panel: Spearman's rank correlation coefficients. The stars indicate significant correlations (* $p < 0.05$; ** $p < 0.01$; *** $p < 0.001$). Background colours indicate the degree of correlation.

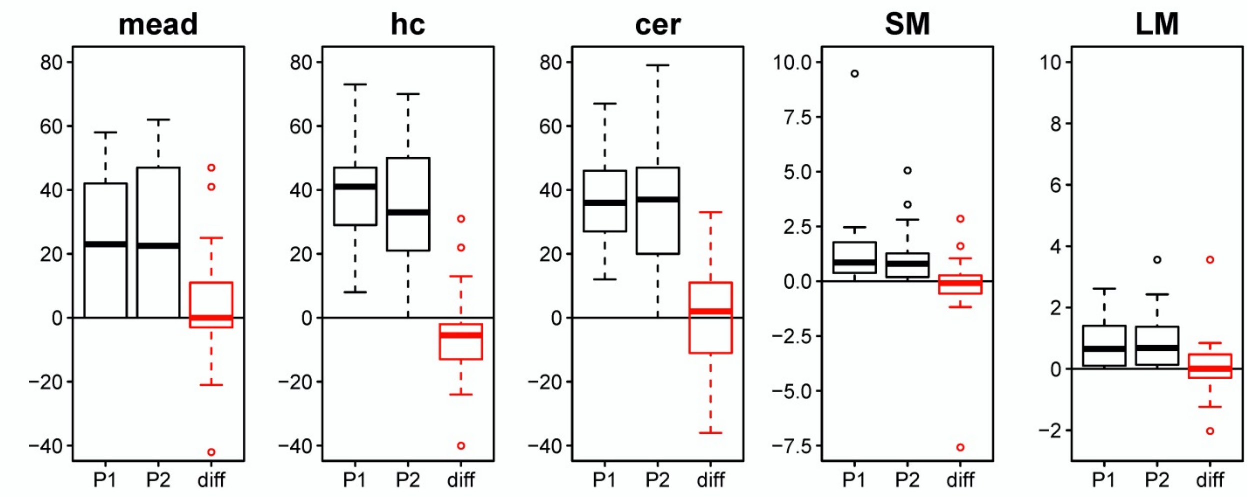

Fig. S3. Management data summarised for the two periods 1985-99 (P1) vs. 2000-2014 (P2) and the respective differences (diff). The boxplots display the means per monitoring site for P1 and P2 and the differences P2-P1 per site (N=22) for the percentages of years featuring meadows (mead), cereals (cer), and so-called hoe crops (hc; includes maize, rape, beets, and potatoes) as main crop and the mean annual inputs of solid (SM) and liquid (LM) farmyard manure (t dry matter ha^{-1} yr^{-1})

SI2: Additional Figures for Results & Discussion

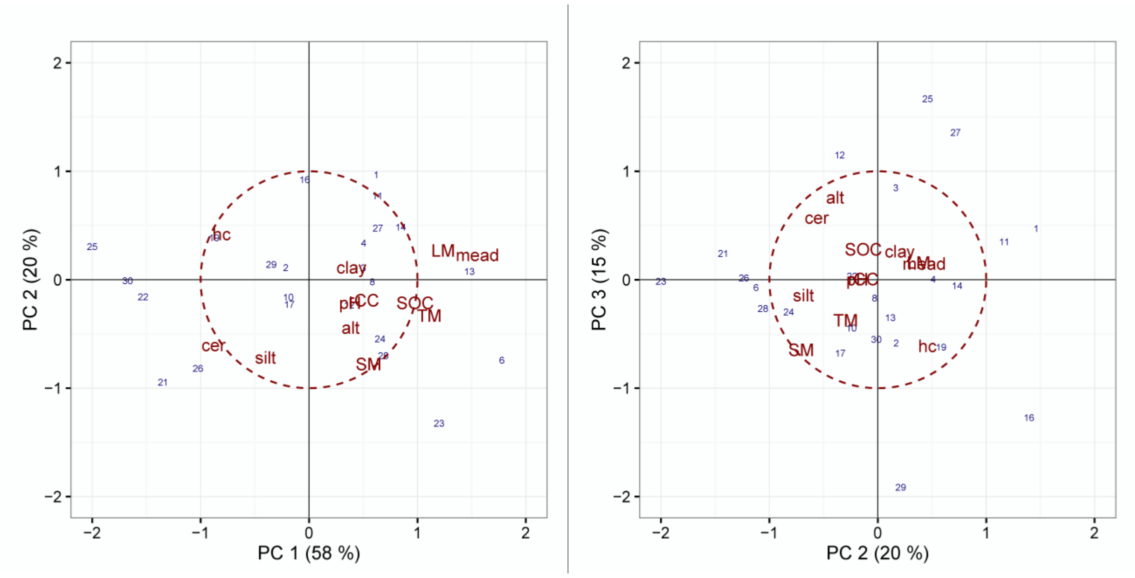

Fig. S4. Correlation biplots for a robust principal component analysis (ROBPCA) for sites with complete data (N=24). Mean values of all sampling campaigns (1990-2014) were used, data transformations are described in the Methods section of the main text. Red labels indicate the loadings of the variables: soil organic carbon content (SOC), altitude (alt), pH, contents of clay and silt, ratio of SOC:clay (rCC), mean annual inputs of farmyard manure in total (TM) and for solid (SM) and liquid (LM) manure separately, and percentages of years (1985-2014) featuring meadows (mead), cereals (cer), and so called hoe crops (hc; includes maize, rape, beets, and potatoes) as main crop, Blue labels indicate the scores of the observations (site ID).

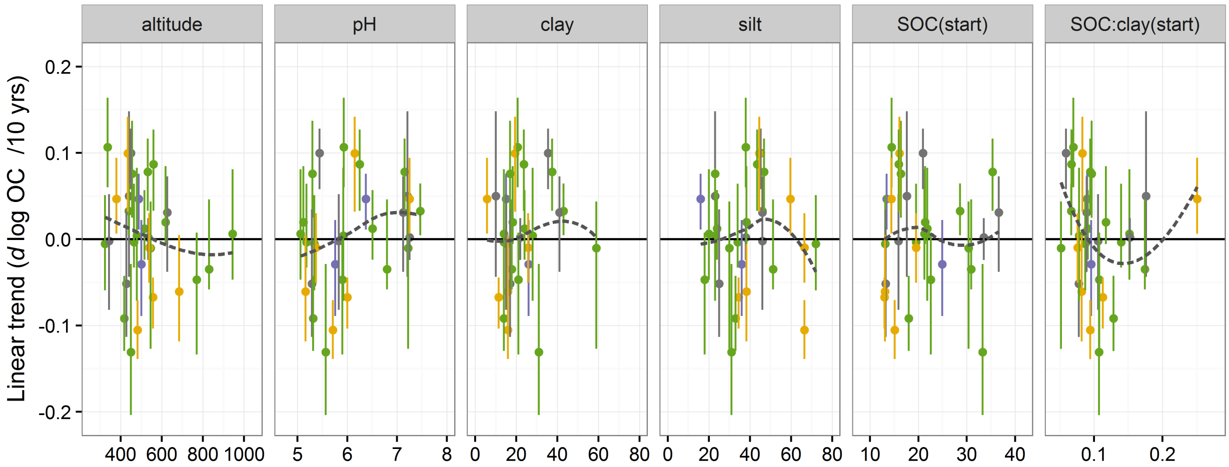

Fig. S5. Trends 1990-2014 of organic carbon (OC) contents per site (slopes of $\log_{10}$(OC); displayed are median [points] and 0.025 to 0.975 quantiles [bars] of bootstrap samples, c.f. main text for details) versus site characteristics, namely altitude (meters above sea level), pH ($CaCl_2$), apparent density of fine earth (AD, mass of fine earth [< 2 mm] per total soil volume, g cm^{-3}), contents of clay and silt (% of fine earth), and OC contents at the beginning of the assessed period (g kg^{-1}). Orange symbols represent permanent cropland sites (N=6), green symbols represent sites with cropland-meadow rotations (N=16), purple symbols represent two sites converted into permanent grassland around 2005 (sites 5, 23), and grey symbols represent sites without management data (N=6). Broken line: LOESS smoother for span = 1.

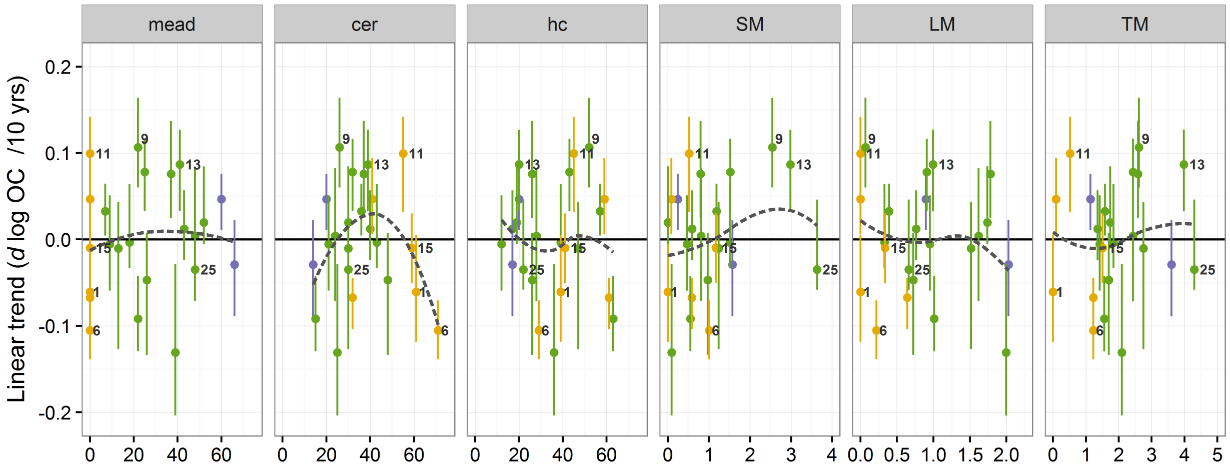

Fig. S6. Trends 1990-2014 of organic carbon (OC) contents per site (slopes of log_{10}(OC); displayed are median [points] and 0.025 to 0.975 quantiles [bars] of bootstrap samples, c.f. main text for details) versus management (average values 1985-2014), namely the percentages of years featuring meadows (mead), cereals (cer), and hoe crops (hc; includes maize, rape, beets, and potatoes) as main crop, and mean annual inputs of solid (SM) and liquid (LM) farmyard manure (t dry matter ha^{-1} yr^{-1}). Orange symbols represent permanent cropland sites (N=6), green symbols represent sites with cropland-meadow rotations (N=16), and purple symbols represent two sites converted into permanent grassland around 2005 (sites 5, 23). Broken line: LOESS smoother for span = 1.

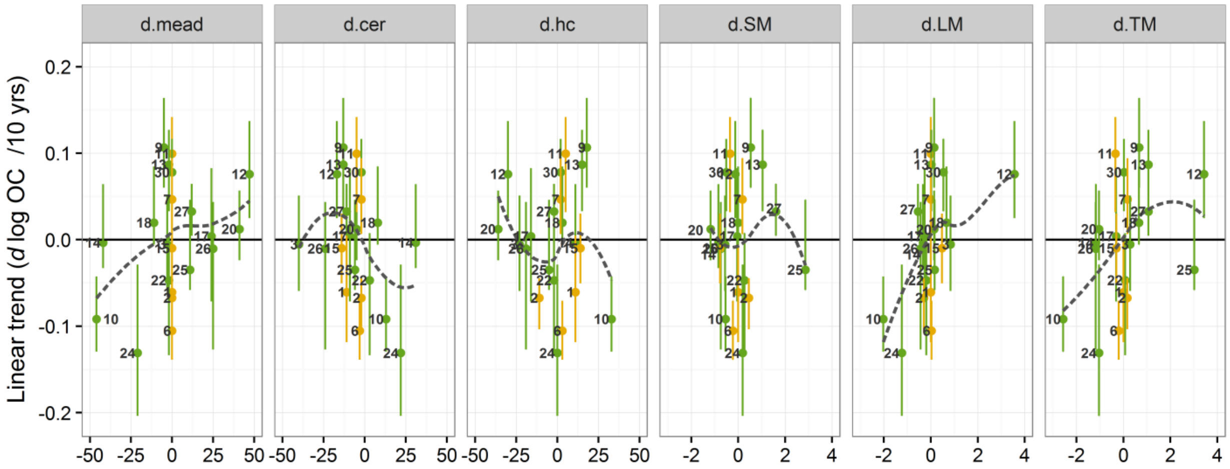

Fig. S7. Trends 1990-2014 of organic carbon (OC) contents per site (slopes of log_{10}(OC); displayed are median [points] and 0.025 to 0.975 quantiles [bars] of bootstrap samples, c.f. main text for details) versus differences in management 1985-99 (P1) vs. 2000-14 (P2), namely the differences P2-P1 in percentages of years featuring meadows (d.mead), cereals (d.cer), and hoe crops (d.hc; includes maize, rape, beets, and potatoes) as main crop, and the differences P2-P1 in mean annual inputs of solid (d.SM) and liquid (d.LM) farmyard manure (t dry matter ha^{-1} yr^{-1}). Orange symbols represent permanent cropland sites (N=6), green symbols represent sites with cropland-meadow rotations (N=16), and purple symbols represent two sites converted into permanent grassland around 2005 (sites 5, 23). Labels: ID of selected sites. Broken line: LOESS smoother for span = 1.

SI3: Minimum detectable change (MDC) and variability

The aim of monitoring programs is to detect temporal trends. Dependent on their settings, changes in soil properties are captured with varying reliability. Hence, the minimum detectable change (MDC) is an important criterion of quality (Smith 2004). Generally, repeated sampling of the same set of well defined sites is considered more efficient than sampling different sites each time (Lark 2009). The regional mean change Y is estimated from the changes at the individual sites, e.g., using a hierarchical model. Estimates of Y may be considered realisations of a random variable with mean μ_y (the 'true' change) and variance proportional to (i) the spatial variability of the SOC change (but not the spatial variability of the SOC itself), (ii) errors due to sampling, sample preparation, and chemical analyses, (iii) variance caused by relocation errors, and (iv) variance due to short-term variations in SOC (and soil properties in general). Over the short term, SOC fluctuates due to seasonal patterns and/or random processes (e.g., rainfall) regardless of the long-term evolution. In contrast to the other error sources mentioned, short-term variability has only been recognised by a few researchers. For instance, Leinweber et al. (1994) reported variations of up to 40 g SOC kg^{-1} (representing 15 % relative change) within one year for a single field, and Wuest (2014) reported relative variations of 14-16 % within 39 months. From a long-term perspective, short-term variability represents noise and hampers the detection of long-term trends.

Methods

The MDC of the NABO monitoring program was assessed by conducting a power analysis for simulated datasets with varying temporal SOC trends. The (log-transformed) SOC data of five sampling campaigns from 1990 to 2014 served as a basis for the simulations. First, the slopes per site (linear trend) were extracted and centred giving a mean of 0 to represent the population $\hat{S} = \{\hat{S}_1, \hat{S}_2, \ldots, \hat{S}_N\}$ of potential slopes. $\hat{S}$ reflects the variability of linear trends between sites (term σ^2_Y in Equation 1). Second, we derived the deviations $\widehat{D}$ of the individual samplings from the linear trends obtained. $\widehat{D}$ reflects the variance introduced both by short-term variations and the various error sources (remaining terms in Equation 1). We simulated datasets containing 30 sites with 7 samplings separated by five years as follows: a) we drew a bootstrap sample of size 30 from $\hat{S}$, yielding an individual slope per hypothetical site *i;* b) for each site *i*, we drew a bootstrap sample of size 7 from $\widehat{D}$ representing the residuals of 7 samplings; c) we combined the data of steps a and b to receive a dataset X_0 without a global trend Y; d) we derived the datasets X_1, X_2, … with varying global trends Y by adding a relative increase of 1, 2, 2.5, 3, …, 6, 7, 8 % per 10 years to $X_{0;}$ e) we subsequently derived datasets with varying observation periods by retaining data from two, three… seven sampling campaigns; f) we tested for a linear trend (α = 0.05) by fitting a linear-mixed model or, for datasets including only two sampling campaigns, by performing a paired t-test. Steps a to f were iterated 500 times. Then, the proportion of iterations where the model correctly identified a linear trend corresponded to the power (i.e., the probability of detecting an inherent trend). The smallest simulated SOC trend where the power ≥ 0.8 was considered the MDC. Similarly, we assessed the MDC of datasets with less/more monitoring sites by repeating the same procedure for simulated datasets containing 20, 60, and 100 sites.

We assessed the variability of the replicate samples per site and sampling by calculating the sample standard deviation *s* of the log-transformed data (natural logarithm). Equally, the variability of the repeated samplings per site was assessed by calculating *s* for the deviations of the individual samplings from the linear trends (this may be considered an estimate of the minimum variability between samplings). For easier comparison with other reports,

we additionally estimated the coefficient of variation (CV) defined as *s* (of the original, untransformed data) divided by their mean. For the sake of simplicity, we estimated CV by $CV(x_{1..N}) \approx s(\log [x_{1..N}])$ being appropriate for CV up to about 0.25.

Results & Discussion

After five sampling campaigns (time points), a monitoring scheme according to NABO is able to detect a relative global SOC change provided that it is at least 0.35 % per year (Table S1, Fig. S8, Fig. S9). This roughly corresponds to a 7 % relative change after 20 years. The inclusion of additional time points makes no further substantial improvement to the MDC, but using fewer time points increases it. Using two or three time points makes the long-term trends barely detectable because they are confounded with artefacts and short-term variations in SOC; this causes an elevated risk of false positives (type I error: "the test indicates a significant change although there is none"). The MDC of course reflects the probability of false negatives (type II error: "there is a real trend but it cannot be seen"), which reflects the sensitivity of our system. Various factors influence MDC, as discussed above. Although relocation errors seem negligible with our monitoring system due to the high accuracy of relocation, further sources of variance are briefly discussed below.

Table S1 Minimum detectable change (MDC; rounded to 0.05) for varying numbers of monitoring sites and observation periods. The case of 30 sites (shaded column) represents the monitoring scheme of the Swiss Soil Monitoring Network NABO.

Number of monitoring sites	20	30	60	100
km^2 /site	210	140	70	40
5 years (2 time points)	0.90	0.80	0.50	0.40
10 years (3 time points)	0.60	0.45	0.35	0.25
15 years (4 time points)	0.45	0.40	0.30	0.20
20 years (5 time points)	0.45	0.35	0.25	0.20
25 years (6 time points)	0.45	0.35	0.25	0.20
30 years (7 time points)	0.45	0.35	0.25	0.20

Comparing SOC across individual replicates per sampling for each site (variability within sampling: V_w) and across repeated samplings per site (variability between samplings: V_b) highlighted differences between sites (SI.5 for detailed results). The standard deviation *s* (of the $\log_{10}$-transformed SOC) of the four replicates per site and sampling captures the overall variance of sampling, sample preparation, and chemical analyses. For our study, *s* pooled per site (s_p) ranged from 0.006 to 0.027 with mean 0.014 and median 0.013 (Fig. S10; or expressed as CV: 1.3 to 6.1 %, mean 3.2 %, median 3.0 %). There was no correlation between s_p and SOC content, meaning that the relative variability was constant for the whole concentration range of 10 to 40 g kg^{-1}. In addition, the variance within samplings fluctuated slightly between sampling campaigns, but there was no consistent trend over time (Fig. S11); hence, the variability of the replicates did not change over time. The variability between samplings at the same site was expressed as *s* for the residuals relative to the linear regression line. For our sites, *s* ranged from 0.005 to 0.039 with mean 0.018 and median 0.014 (Fig. S12; or expressed as CV: 1.1 to 8.9 %, mean 4.1 %, median 3.3 %). The variability between samplings observed for individual sites was correlated neither with SOC content nor with s_p (the variability within individual samplings; Fig. S13). Hence, we assume that short-term variability of SOC was the dominant factor for V_b, while further sources of error were largely balanced out by averaging over four replicates.

In consequence, short-term variability of SOC, and thus V_b, cannot be estimated from data from a single survey. Similarly, spatial variability of SOC trends can only be estimated from repeated samplings. Because these two factors strongly influence MDC, the same applies for estimating MDC. However, to our best knowledge, only one previous study used data from resampled sites: for England and Wales, the MDC for croplands was estimated at 2 g kg^{-1} using two time points separated by 12 years (Saby et al. 2008). This corresponded roughly to a 7 % relative change in total or 0.6 % per year, in line with the MDCs reported in Table S1 (England & Wales: one site per 73 km^2).

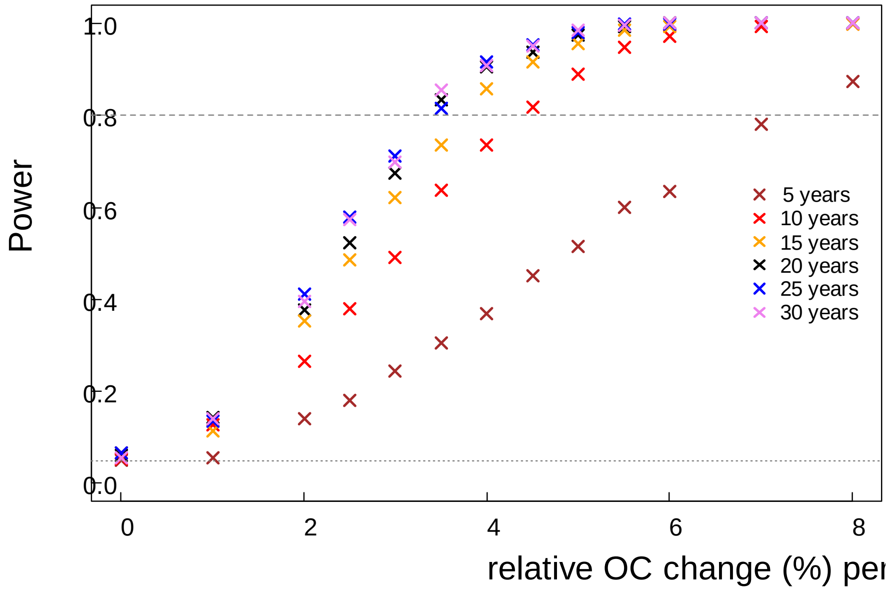

Fig. S8. Power of detecting a relative change in organic carbon (OC) for data including varying numbers of sampling campaigns (separated by five years each) and thus varying periods of observation within a monitoring scheme including 30 cropland sites using the methodology applied by the Swiss Soil Monitoring Network NABO and using the variances (within sites and between sites) observed for monitoring sites in Swiss cropland.

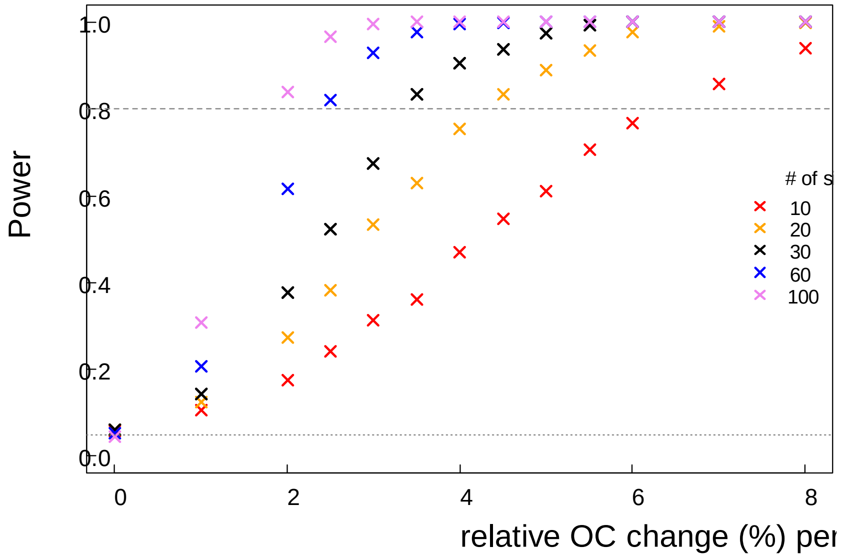

Fig. S9. Power of detecting a relative change in organic carbon (OC) for data from five sampling campaigns (separated by five years each) within a hypothetical monitoring scheme including 10, 20, 30, 60, or 100 cropland sites using the methodology applied by the Swiss Soil Monitoring Network NABO and using the variances (within sites and between sites) observed for monitoring sites in Swiss cropland.

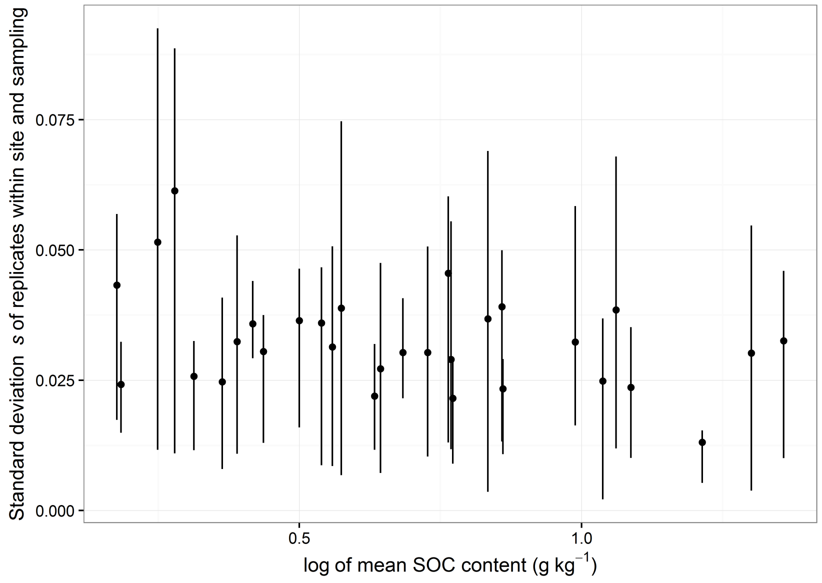

Fig. S10. Variability within individual samplings per site: standard deviations *s* of organic carbon (OC) contents of the replicate samples within site and sampling versus the mean SOC content (over all samplings) of the site. The lines indicate the range of s observed for the various samplings at the individual sites, the dots indicate the pooled *s* per site.

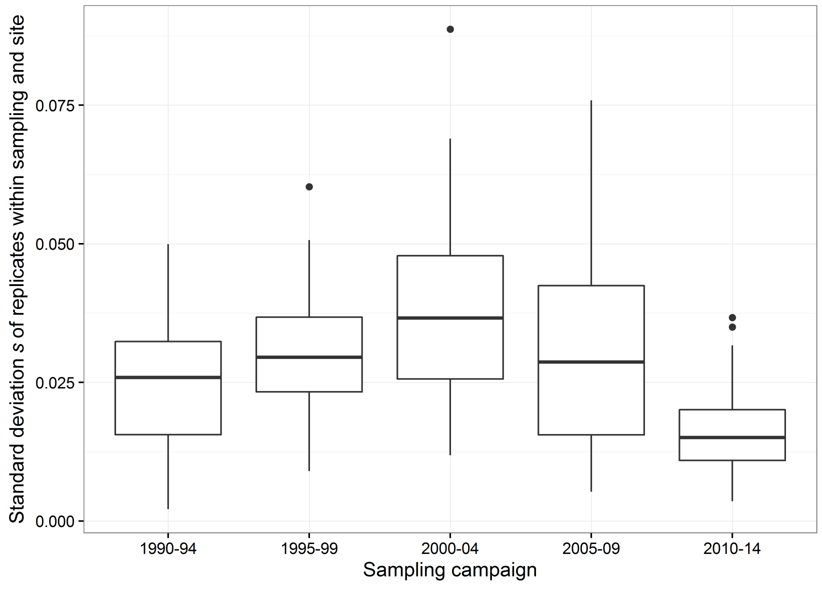

Fig. S11. Variance within individual samplings per site: boxplot of standard deviations *s* of soil organic carbon contents of the replicate samples per site and sampling versus sampling campaign.

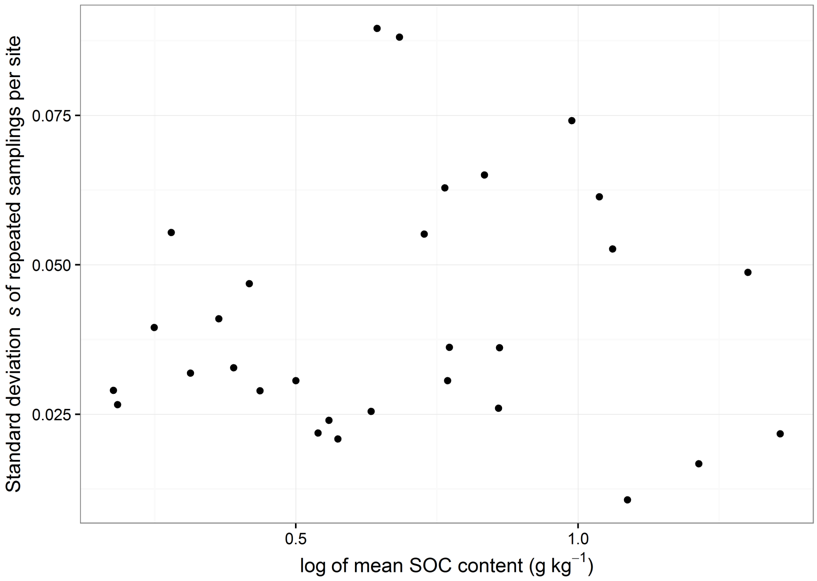

Fig. S12. Variability between samplings of individual sites: standard deviations *s* of organic carbon (SOC) contents of the repeated samplings per site versus the mean SOC content (over all samplings) of the site.

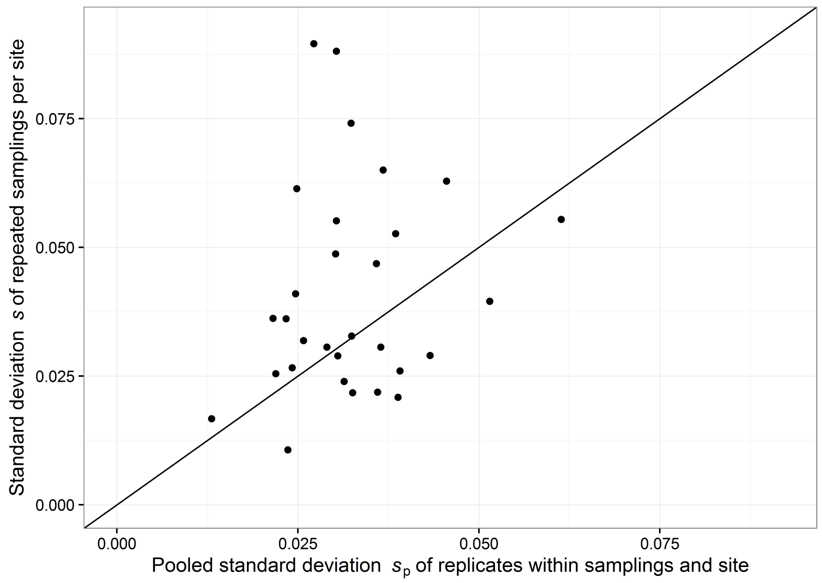

Fig. S13. Standard deviations *s* of organic carbon contents of the repeated samplings per site versus *s* of OC contents of the replicate samples within site and sampling.

References

Lark, R. M. (2009). Estimating the regional mean status and change of soil properties: two distinct objectives for soil survey. *European Journal of Soil Science*, *60*(5), 748–756. doi:10.1111/j.1365-2389.2009.01156.x

Leinweber, P., Schulten, H.-R., & Körschens, M. (1994). Seasonal variations of soil organic matter in a long-term agricultural experiment. *Plant and Soil*, *160*(2), 225–235. doi:10.1007/BF00010148

Saby, N. P. A., Bellamy, P. H., Morvan, X., Arrouays, D., Jones, R. J. A., Verheijen, F. G. A., et al. (2008). Will European soil-monitoring networks be able to detect changes in topsoil organic carbon content? *Global Change Biology*, *14*(10), 2432–2442. doi:10.1111/j.1365-2486.2008.01658.x

Smith, P. (2004). How long before a change in soil organic carbon can be detected? *Global Change Biology*, *10*(11), 1878–1883. doi:10.1111/j.1365-2486.2004.00854.x

Wuest, S. (2014). Seasonal Variation in Soil Organic Carbon. *Soil Science Society of America Journal*, *78*(4), 1442. doi:10.2136/sssaj2013.10.0447
